# Post-COVID Condition in Adults and Children Living in the Same Household in Italy: A Prospective Cohort Study Using the ISARIC Global Follow-Up Protocol

**DOI:** 10.3389/fped.2022.834875

**Published:** 2022-04-21

**Authors:** Danilo Buonsenso, Daniel Munblit, Ekaterina Pazukhina, Antonia Ricchiuto, Dario Sinatti, Margherita Zona, Alessia De Matteis, Federico D’Ilario, Carolina Gentili, Roberta Lanni, Teresa Rongai, Patrizia del Balzo, Maria Teresa Fonte, Michele Valente, Giuseppe Zampino, Cristina De Rose, Louise Sigfrid, Piero Valentini, FIMP-Roma, Ilaria Sani

**Affiliations:** ^1^Department of Woman and Child Health and Public Health, Fondazione Policlinico Universitario A. Gemelli IRCCS, Rome, Italy; ^2^Global Health Research Institute, Istituto di Igiene, Università Cattolica del Sacro Cuore, Rome, Italy; ^3^Sezione di Microbiologia, Dipartimento di Scienze Biotecnologiche di Base, Cliniche Intensivologiche e Perioperatorie, Università Cattolica del Sacro Cuore, Rome, Italy; ^4^Department of Paediatrics and Paediatric Infectious Diseases, Institute of Child’s Health, I.M. Sechenov First Moscow State Medical University, Sechenov University, Moscow, Russia; ^5^Inflammation, Repair and Development Section, Faculty of Medicine, National Heart and Lung Institute, Imperial College London, London, United Kingdom; ^6^Laboratory of Health Economics, Institute of Applied Economic Studies, Russian Presidential Academy of National Economy and Public Administration, Moscow, Russia; ^7^Center for Advanced Financial Planning, Macroeconomic Analysis and Financial Statistics, Financial Research Institute, Ministry of Finance of the Russian Federation, Moscow, Russia; ^8^Pediatra di Libera Scelta, Federazione Italiana Medici Pediatri, Rome, Italy; ^9^International Severe Acute Respiratory and Emerging Infection Consortium Global Support Centre, Centre for Tropical Medicine and Global Health, Nuffield Department of Medicine, University of Oxford, Oxford, United Kingdom

**Keywords:** COVID-19, long COVID, post-COVID “long-haulers”, children, adults

## Abstract

**Background:**

Emerging evidence shows that both adults and children may develop post-acute sequelae of SARS-CoV-2 infection (PASC). The aim of this study is to characterise and compare long-term post-SARS-CoV-2 infection outcomes in adults and children in a defined region in Italy.

**Methods:**

A prospective cohort study including children (≤18 years old) with PCR-confirmed SARS-CoV-2 infection and their household members. Participants were assessed via telephone and face-to-face visits up to 12 months post-SARS-CoV-2 diagnosis of household index case, using the ISARIC COVID-19 follow-up survey.

**Results:**

Of 507 participants from 201 households, 56.4% (286/507) were children, 43.6% (221/507) adults. SARS-CoV-2 positivity was 87% (249/286) in children, and 78% (172/221) in adults. The mean age of PCR positive children was 10.4 (SD = 4.5) and of PCR positive adults was 44.5 years (SD = 9.5), similar to the PCR negative control groups [children 10.5 years (SD = 3.24), adults 42.3 years (SD = 9.06)]. Median follow-up post-SARS-CoV-2 diagnosis was 77 days (IQR 47–169). A significantly higher proportion of adults compared to children reported at least one persistent symptom (67%, 68/101 vs. 32%, 57/179, *p* < 0.001) at the first follow up. Adults had more frequently coexistence of several symptom categories at both follow-up time-points. Female gender was identified as a risk factor for PASC in adults (*p* 0.02 at 1–3 months and *p* 0.01 at 6–9 months follow up), but not in children. We found no significant correlation between adults and children symptoms. In the paediatric group, there was a significant difference in persisting symptoms between those with confirmed SARS-CoV-2 infection compared to controls at 1–3 months follow up, but not at 6–9 months. Conversely, positive adults had a higher frequency of persisting symptoms at both follow-up assessments.

**Conclusion:**

Our data highlights that children can experience persistent multisystemic symptoms months after diagnosis of mild acute SARS-CoV-2 infection, although less frequently and less severely than co-habitant adults. There was no correlation between symptoms experienced by adults and children living in the same household. Our data highlights an urgent need for studies to characterise PASC in whole populations and the wider impact on families.

## Introduction

Despite more than 2 years since the first description of novel coronavirus disease 2019 (COVID-19), the world is still in the middle of the pandemic. During this time, however, advances have been made in the understanding of the acute disease, its management and treatment and effective vaccines have been developed in a historically short timeframe. (1, 2). Whereas advancements have been made to improve acute patient outcomes, the evidence into longer term consequences of SARS-CoV-2 infection on physical and psychological health and impact on daily living is still limited (3).

Growing evidence is showing that a significant percentage of adults develop long-lasting post-SARS-CoV-2 sequelae, such as fatigue, headache, chest pain, dyspnoea, neurological, psychological, and cardiovascular symptoms, with impact on occupation and quality of life. Although the aetiology has not been identified, there is increasing evidence that persistent infection, latent virus, chronic inflammation, autoimmunity, endothelial dysfunction, lung and brain perfusion defects, and/or autonomic dysfunction may play a role in this complex clinical picture (3–6), defined as Post-Acute Sequelae of SARS-CoV-2 infection (PASC), post-COVID condition or Long COVID. Although the World Health Organisation (WHO) together with the global health community have developed a definition for post-COVID condition in adults, there is still no case definition for children, and no effective treatment identified. There is no consensus on supportive care and limited funding for interventional studies available.

The recent WHO definition for adults’ states that post COVID-19 condition occurs in individuals with a history of probable or confirmed SARS-CoV-2 infection, usually 3 months from the onset of COVID-19 with symptoms that last for at least 2 months and cannot be explained by an alternative diagnosis. Common symptoms include fatigue, shortness of breath, cognitive dysfunction but also others, and generally have an impact on everyday functioning. Symptoms may be new onset following initial recovery from an acute COVID-19 episode or persist from the initial illness. Symptoms may also fluctuate or relapse over time.” Importantly, the WHO specify that “a separate definition may be applicable for children” (7). In fact, although children have been relatively spared from severe acute COVID-19 disease compared to adults (8), emerging studies suggest that children can also suffer from PASC (9–14), although its prevalence is still unclear (9–14).

So far studies aiming to define PASC have either only enrolled adults or children with SARS-CoV-2 infection but cohorts including both populations are lacking, limiting the possibility to compare prevalence, clinical characteristics of and risk factors for developing PASC across age spectra (15). This study aims to investigate and compare the prevalence and characteristics of PASC in adults and children living in the same households in Rome, Italy, by using a, standardised data collection form. The objective is to inform health service and public health policies, and further studies to improve long term COVID-19 outcomes.

## Materials and Methods

### Study Design, Setting, and Participants

This is a prospective cohort study including children diagnosed with SARS-CoV-2 and their household contacts. Children (≤18 years old) diagnosed with SARS-CoV-2 infection using reverse transcriptase polymerase chain reaction (RT-PCR) between April 1st, 2020 and April 31st, 2021 at the Department of Women and Child Health of the Fondazione Policlinico Universitario A. Gemelli IRCCS of Rome, Italy and their household members were invited to participate. The Institution is a regional referral COVID-19 centre for adults and also has a dedicated Paediatric Infectious Disease In-patient Unit and an outpatient Paediatric Post-COVID Unit using the ISARIC survey as a screening tool for persisting symptoms (16). Therefore, both children hospitalised and community patients assessed in the outpatient unit were included.

All members of the household were tested for SARS-CoV-2 using RT-PCR as part of contact-tracing. Household contacts were tested within 48 h after the suspected index cases. If negative they were tested again after 10 days, or if they developed symptoms at time of symptom onset, as per Italian contact-tracing procedures (17). Those that remained negative and did not develop acute symptoms during the incubation period were included as a negative control cohort.

The parents of the children (household index case) were contacted by paediatric residents between September 30, 2020 and June 1, 2021. The follow-up assessments were conducted *via* an interview with the parents/carers, by phone call or during a face-to-face outpatient assessment. The first assessment was made 1–3 months, followed by a second assessment 3–6 months post-SARS-CoV-2 diagnosis of the index case. This is a longitudinal study including positive index cases over a period of time for serial follow-up interviews every 3 months. A subgroup of the participants have been assessed twice at the time of the writing of this manuscript. Non-responders were contacted by telephone three times before being considered lost to follow-up.

We used the ISARIC Global COVID-19 follow up protocol for adults and children (18) and associated standardised data collection forms. The assessment methodology has been presented in an earlier linked study (9, 10). Information about the participants current health status was assessed using the ISARIC COVID-19 Health and Wellbeing Follow-Up Survey for Children (version 1.0 translated into Italian). The survey assesses physical and psychosocial health and wellbeing, and impact on daily functioning, behaviour, relationships and daily living (16). The survey documents data on demographics, pre-existing comorbidities, acute severity, information on the acute phase of the disease (symptoms, comorbidities, and clinical outcomes), severity (hospital admission, paediatric intensive care (PICU/ICU), oxygenation). Moreover, data on readmissions, parental perception of changes in their child’s emotional and behavioural status, including reasons for observed changes (direct or indirect impact of COVID-19 or both), persisting symptoms at the follow-up assessment, and overall health condition compared to prior to the index case SARS-CoV-2 diagnosis, and mortality (16).

For adult household members we used the aligned ISARIC COVID-19 follow-up survey for adults (18, 19). Similarly, to the paediatric survey it documents data on the presence of persisting or new symptoms not present before SARS-CoV-2 infection onset, and on psychological health and impact on daily living using standardised tools. Since the new definition of Post-COVID condition in adults was released after the study was designed, we decided to describe all symptoms lasting more than 1 month in both adults and children, but providing a specific analysis of symptoms persisting after 3 months post-SARS-CoV-2 infection, to characterise PASC according to the latest WHO definition (7). Although this definition is not specific for children, given the current lack of a paediatric definition, we applied the same for patients younger than 18 years of age.

### Outcomes

The *primary aim* of this study is to characterise cluster of persisting symptoms in a cohort of children post-SARS-CoV-2 infection in Rome, Italy.

*Secondary objectives are:*

- To characterise and compare the clusters of persisting symptoms in children and adults living in the same household, post-SARS-CoV-2 infection.
- To compare the patterns of symptoms in children and adults post-SARS-CoV-2 infection with negative household controls.
- To determine if the presence of persisting symptoms in adults correlates with the presence of persisting symptoms in children living in the same household.
- To identify risk factors for developing post-acute SARS-CoV-2 sequelae in adults and children.

### Ethical Approval

This study was approved by the Ethics Committee of the Fondazione Policlinico Universitario A. Gemelli IRCCS of Rome (ID3777). Written informed consent was obtained by each parent/carer/guardian for both their own participation and their children’s, and from the child as well when older than 12 years of age. Consent was obtained either during an outpatient visit or by contacting the families via a phone call, where the study procedures were explained, followed by a consent form emailed to the participants, and returned to the study staff.

### Exposure and Outcome Variables

For the purpose of this study, we defined “persistent symptoms” as symptoms present at the time of the follow-up interview. that had been present for at least 4 weeks since diagnosis. These were sub-categorised into respiratory, neurological, sensory, sleep, gastrointestinal, general (including headache, malaise, and fatigue), dermatological, cardiovascular, urogenital, and musculoskeletal, informed by previously published literature and ISARIC Global Paediatric COVID-19 follow-up working group consensus discussions (20). Health status was assessed using the EuroQol overall health status tool (21), where zero is categorised as the worst possible health and 100 the best possible health.

### Statistical Analysis

Descriptive statistics were calculated for baseline characteristics. Continuous variables were summarised as mean (SD), median (with interquartile range) and categorical variables as frequency (percentage). The two-sided Student *t*-test was used for testing hypotheses on differences in proportions between groups, with two-sided *p*-value of <0.05. Paired *t*-test of two-sided *p*-value of <0.05 was used to evaluate persistence of symptoms among individuals across the two follow-up periods. Student’s *t*-test assumptions were met in order to compare proportions between groups. Bootstrapped confidence intervals and correlation coefficients were used to compare persisting symptoms in adults and children from the same households.

We performed multivariate logistic regression to investigate associations of demographic characteristics, comorbidities (limited to those reported in at least 5% of participants), and hospitalisation during acute infection with persistent symptom present at the time of the follow-up assessment. We included all participants for whom the variables of interest were available for the final analysis, without imputing missing data. The differing denominators used indicate missing data. Odds ratio (OR) and s ratios were calculated together with 95% confidence intervals (CIs). We evaluated three model for long COVID-19 of the following specifications: for children, for children and their adult relatives with previous hospitalisation covariate (estimated on 375 observations), for children and their adult relatives without previous hospitalisation covariate and age categories (estimated on 634 observations). Age categorisation was decided given a gradient of more severe COVID-19 with increasing age.

Upset plots were used to present the co-existence of persistent symptom categories. Two-sided *p*-values were reported for all statistical tests, a *p*-value below 0.05 was considered to be statistically significant. Statistical analysis was performed using R version 4.0.2. Packages used included dplyr, ggplot2, ComplexUpset.

### Patient and Public Involvement

The survey was developed by the ISARIC Global Paediatric and Adult COVID-19 follow-up working groups and informed by a wide range of global stakeholders with expertise in infectious diseases, critical care, paediatrics, epidemiology, allergy-immunology, respiratory medicine, psychiatry, psychology and methodology and patient-public representatives including people living with Long COVID, members of the Long COVID Support group. The data collection forms were distributed to the members of the patient groups and suggestions from parents/carers were implemented to ensure key symptoms and sequelae were assessed.

## Results

### Study Population

Of 507 participants 56.4% (286/507) were children, 43.6% (221/507) adults. Of these, 87% (249/286) of children and 78% (172/221) of adults were SARS-CoV-2 PCR positive (Table 1). The median age of the paediatric PCR+ group was 11.1 years (IQR, 6.7–13.6; range, 0.4–18.8 years), 51.4% were females. Median follow-up time post-SARS-CoV-2 diagnostic testing was 77 days (IQR 47–169).

**TABLE 1:** Study population.

		Children		Adults	
			
		RT-PCR positive (*n* = 249)	RT-PCR negative* (*n* = 37)	RT-PCR positive (*n* = 172)	RT-PCR negative* (*n* = 49)
Age (mean, std)	1–3 months follow-up	10.1 (4.30)	10.5 (3.24)	44.5 (8.26)	42.3 (9.06)
	6–9 months follow-up	10.6 (4.65)		44.6 (10.3)	
Number of participants (*n*)	1–3 months follow-up	179	37	101	49
	6–9 months follow-up	138		107	
Gender (% females)	1–3 months follow-up	48.9	52.6	44.0	46.3
	6–9 months follow-up	61.5		45.7	

*RT-PCR, real-time polymerase chain reaction; Std, standard deviation.*

*The timeframe is calculated from the date the index case was diagnosed.*

*All family members were assessed at the same time point.*

*Timepoints in the negative PCR groups have not been included since no starting point of a SARS-CoV-2 infection present; they were interviewed at the same time of the positive household members.

*The average size of the households were 2.5 members. The average infection rate per household was 87% among children and 80% among adults.*

Most children (72%, 179/249) were interviewed 1–3 months post-SARS-CoV-2 testing, 55% (138/249) after 6–9 months and 9% (22/249) after 12 months. About a third (27.7%, 69/249) were assessed twice at 1–3 and 6–9 months (Table 1). The most common pre-existing conditions are provided in the Supplementary Table 1, while main demographic data of included adults and children during different timepoints are reported in Table 1.

During the acute infection, 2.4% (6/249) of children were admitted to hospital, of these 33.3% (2/6) required PICU admission. Amongst adults, 12.2% (21/172) were hospitalised, of which, 4.8% (1/21) were admitted to ICU. Symptoms experienced in the first 14 days of COVID-19 illness by children with microbiologically confirmed infection are reported in the Supplementary Table 2.

### Persisting Symptoms

At the time of the follow-up assessment a significantly higher proportion of adults compared to children with previous documented SARS-CoV-2 infection reported at least one persistent symptom (67%, 68/101 vs. 32%, 57/179, *p* < 0.001). The prevalence of the most commonly reported persisting symptoms by children and adults at the 1–3 months and 6–9 months time points are presented in Table 2. Overall, children had a higher probability of being fully recovered compared to adults at both the 1–3 months (*p* = 0.001) and 6–9 months follow-up assessments (*p* = 0.01). At the 1–3 months assessment adults had statistically significant higher probability of experiencing asthenia (*p* < 0.0001), muscle pain (*p* < 0.017), rash (*p* = 0.01), and pain on breathing (*p* = 0.04). At the 6–9 months assessment, adults had a statistically significant higher probability of reporting muscle and joint pain (*p* < 0.0001), ageusia and anosmia (*p* < 0.0001), pain on breathing (*p* = 0.002), palpitations (*p* = 0.01), diarrhoea (*p* = 0.02), difficulty moving (*p* = 0.01), and joint swelling (*p* = 0.04).

**TABLE 2:** Comparison of proportion of symptoms between adults and children with PCR – confirmed SARS-CoV-2 infection.

Proportion of a symptom/state mean (SD)	1–3 months follow-up			6–9 months follow-up		
		
	Children (*n* = 179)	Adults (*n* = 101)	*P*-value	Children (*n* = 138)	Adults (*n* = 107)	*P*-value
Fully recovered	0.97 (0.18)	0.83 (0.38)	0.001	0.96 (0.2)	0.83 (0.38)	0.016
Feverish	0.43 (0.5)	0.43 (0.5)	0.951	0.12 (0.33)	0.19 (0.39)	0.330
Asthenia	0.2 (0.4)	0.48 (0.5)	0.000	0.14 (0.36)	0.27 (0.45)	0.115
Insomnia	0.24 (0.43)	0.26 (0.44)	0.770	0.19 (0.4)	0.21 (0.41)	0.799
Headache	0.17 (0.38)	0.15 (0.36)	0.634	0.04 (0.2)	0.14 (0.35)	0.051
Muscle pain	0.09 (0.28)	0.21 (0.41)	0.017	0 (0)	0.21 (0.41)	0.000
Joint pain	0.09 (0.28)	0.17 (0.38)	0.090	0 (0)	0.17 (0.38)	0.000
Constipation	0.11 (0.31)	0.1 (0.3)	0.827	0.12 (0.33)	0.08 (0.28)	0.655
Muscle weakness	0.03 (0.18)	0.11 (0.31)	0.037	0.08 (0.27)	0.18 (0.38)	0.127
Cough	0.07 (0.25)	0.1 (0.3)	0.394	0.12 (0.33)	0.07 (0.26)	0.559
Smell	0.05 (0.23)	0.09 (0.29)	0.350	0 (0)	0.14 (0.35)	0.000
Taste	0.07 (0.25)	0.09 (0.29)	0.550	0 (0)	0.12 (0.33)	0.000
Chest pain	0.04 (0.21)	0.1 (0.3)	0.132	0.07 (0.27)	0.04 (0.19)	0.506
Malaise	0.09 (0.28)	0.06 (0.24)	0.467	0 (0)	0.1 (0.31)	0.001
Weight loss	0.04 (0.21)	0.03 (0.17)	0.614	0.12 (0.33)	0.05 (0.21)	0.314
Poor appetite	0.07 (0.25)	0.08 (0.27)	0.709	0.04 (0.2)	0.05 (0.21)	0.850
Rash	0.07 (0.25)	0 (0)	0.013	0.12 (0.33)	0.04 (0.19)	0.250
Abdominal pain	0.1 (0.3)	0.04 (0.2)	0.115	0.04 (0.2)	0.04 (0.19)	0.980
Pain on breathing	0.02 (0.15)	0.09 (0.29)	0.039	0 (0)	0.08 (0.28)	0.002
Palpitations	0.04 (0.21)	0.04 (0.2)	0.894	0 (0)	0.06 (0.23)	0.014
Diarrhoea	0.04 (0.21)	0.04 (0.2)	0.894	0 (0)	0.05 (0.21)	0.025
Difficulty moving	0.02 (0.15)	0.03 (0.17)	0.728	0 (0)	0.06 (0.23)	0.014
Confusion	0.03 (0.18)	0.03 (0.17)	0.908	0 (0)	0.03 (0.17)	0.083
Pins and needles	0 (0)	0 (0)	-	0.04 (0.2)	0.05 (0.21)	0.850
Changes in menstruation	0.03 (0.16)	0 (0)	0.324	0 (0)	0.04 (0.19)	0.083
Joint swelling	0 (0)	0.02 (0.14)	0.158	0 (0)	0.04 (0.19)	0.045
Tremors	0.01 (0.1)	0.01 (0.1)	0.948	0 (0)	0.01 (0.1)	0.320
Erectile dysfunction	0 (0)	0.02 (0.13)	0.321	0 (0)	0.01 (0.11)	0.321
Dizziness	0 (0)	0.01 (0.1)	0.320	0 (0)	0.02 (0.14)	0.158
Dysphagia	0 (0)	0 (0)	–	0 (0)	0.01 (0.1)	0.320
Skin nodules	0 (0)	0 (0)	–	0 (0)	0.01 (0.1)	0.320

**FIGURE 1:**
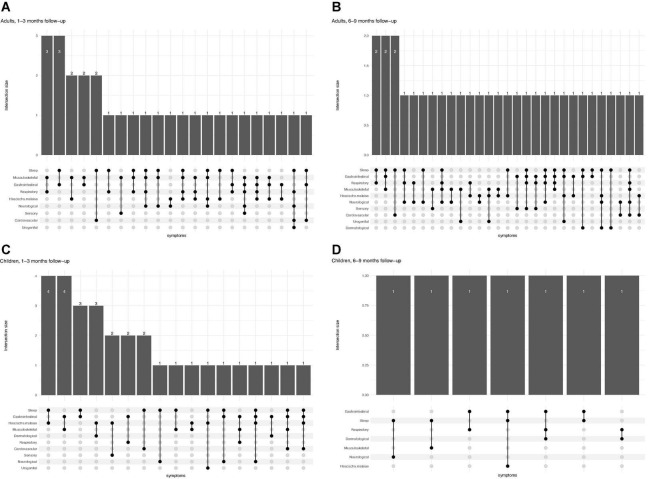
Patterns of multisystem symptoms in adults **(A,B)** and children **(C,D)** at two different time points (1–3 months and 6–9 months follow-up), showing how many patients at each time points have more than one (and which one) symptoms simultaneously.

**FIGURE 2:**
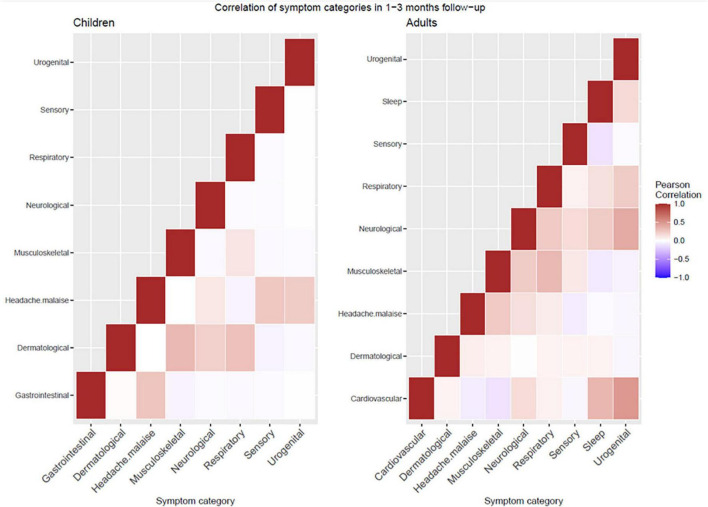
Correlation of symptoms heatmaps in children **(left)** and adults **(right)**.

In particular, 16.7% (30/179) of children experienced concomitant symptoms from at least two different symptom categories at 1–3 months, 3.3% (6/179) experienced symptoms from three or more categories. At 6–9 months follow-up, 5.1% (7/138) experienced persisting symptoms from at least two and 1.4% (2/138) from three or more categories. Adults had a higher frequency of co-existence of several symptom categories at both time points (Figure 1). Correlation heatmaps of reported symptoms are presented in Figure 2.

Table 3 shows frequency of reported clustering of symptoms in children and adults at 1–3 months and 6–9 months follow-up compared to those in the negative cohort. For the paediatric groups, at the 1–3 months follow-up the positive cases reported persistent cardiovascular (*p* = 0.04), dermatological (*p* = 0.01), neurological (*p* = 0.01) symptoms including headache (*p* = 0.04), more frequently compared to controls, but not at the 6–9 months follow-up. Conversely, SARS-CoV-2 positive adults had a statistically significant higher probability of reporting symptoms compared with those that tested negative at both follow-up time points (Table 3). A subset of children (28%, 69/249) and adults (21%, 36/172) were assessed twice, at 1–3- and 6–9-month follow-up. Of these participants, all children experiencing symptoms at 1–3 months (38%, 26/69) had recovered at 6–9 months, while most adults experiencing symptoms at 1–3 months (61%, 22/36) still reported persistence of several problems at 6–9 months (Supplementary Table 3). The impact of gender on symptoms persistence is detailed in Table 4.

**TABLE 3:** Proportion of cluster of symptoms in PCR positive cases and controls.

Proportion of symptom groups mean (SD)	1–3 months follow-up	6–9 months follow-up	RT-PCR negative*	*P*-value NEG vs. POS (1–3 m)	*P*-value NEG vs. POS (6–9 m)
**Children (≤18 years old)**					
Cardiovascular	0.05 (0.21)	–	–	0.04	–
Dermatological	0.04 (0.19)	0.03 (0.15)	–	0.01	0.08
Gastrointestinal	0.16 (0.36)	0.06 (0.23)	0.09 (0.28)	0.19	0.54
Headache	0.12 (0.32)	0.01 (0.09)	–	0.00	0.32
Musculoskeletal	0.09 (0.28)	0.02 (0.12)	0.06 (0.23)	0.49	0.32
Neurological	0.03 (0.15)	0.01 (0.09)	–	0.05	0.32
Respiratory	0.04 (0.19)	0.03 (0.15)	0.03 (0.17)	0.83	0.86
Sensory	0.04 (0.2)	–	–	0.01	n/a?
Sleep	0.24 (0.43)	0.2 (0.41)	0.27 (0.46)	0.83	0.59
Urogenital	0.01 (0.08)	–	0.03 (0.17)	0.44	0.32
Any reported symptom	0.32 (0.47)	0.09 (0.29)	0.19 (0.4)	0.10	0.15
**Adults**					
Bleeding	–	–	–	0.04	0.01
Cardiovascular	0.04 (0.2)	0.06 (0.24)	–	–	0.04
Dermatological	–	0.04 (0.2)	–	0.00	0.00
Gastrointestinal	0.22 (0.42)	0.21 (0.41)	0.05 (0.22)	0.02	0.01
Headache, malaise	0.17 (0.38)	0.18 (0.39)	0.05 (0.22)	0.00	0.00
Musculoskeletal	0.37 (0.49)	0.31 (0.47)	0.08 (0.27)	0.01	0.00
Neurological	0.07 (0.26)	0.14 (0.34)	–	0.00	0.00
Respiratory	0.17 (0.38)	0.15 (0.35)	–	0.00	0.00
Sensory	0.11 (0.32)	0.16 (0.37)	–	0.00	0.01
Sleep	0.26 (0.44)	0.22 (0.42)	0.08 (0.27)	0.32	0.04
Urogenital	0.01 (0.1)	0.04 (0.2)	–	0.00	0.00
Any reported symptom	0.67 (0.48)	0.58 (0.5)	0.2 (0.4)	0.04	0.01

*NEG, negative; POS, positive.*

*Timepoints in the negative PCR groups have not been included since no starting point of a SARS-CoV-2 infection present.

**TABLE 4:** Comparison of symptom proportions by gender stratified by follow-up time period in children and adults.

Proportion of symptom groups mean (SD)	1–3 months follow-up			6–9 months follow-up		
		
	Males	Females	*P*-value	Males	Females	*P*-value
**Children (≤18 years old)**						
Bleeding	–	–	na	–	–	na
Cardiovascular	0.05 (0.21)	0.05 (0.21)	0.97	–	–	na
Dermatological	0.04 (0.19)	0.04 (0.19)	0.99	0.05 (0.21)	–	0.08
Gastrointestinal	0.12 (0.32)	0.2 (0.4)	0.14	0.08 (0.26)	0.04 (0.18)	0.29
Headache, malaise	0.1 (0.31)	0.13 (0.34)	0.62	–	0.02 (0.13)	0.32
Musculoskeletal	0.09 (0.29)	0.08 (0.28)	0.81	0.03 (0.17)	–	0.16
Neurological	0.04 (0.19)	0.02 (0.11)	0.32	–	0.02 (0.13)	0.32
Respiratory	0.03 (0.15)	0.05 (0.21)	0.40	0.05 (0.21)	–	0.08
Sensory	0.04 (0.19)	0.05 (0.21)	0.69	–	–	na
Sleep	0.18 (0.39)	0.3 (0.47)	0.18	0.13 (0.35)	0.3 (0.49)	0.33
Urogenital	–	0.02 (0.11)	0.32	–	–	na
Any symptom	0.27 (0.45)	0.36 (0.49)	0.18	0.13 (0.34)	0.05 (0.22)	0.09
**Adults**						
Bleeding	0 (0)	0 (0)	na	0 (0)	0 (0)	na
Cardiovascular	0.05 (0.23)	0 (0)	0.16	0.05 (0.21)	0.06 (0.23)	0.79
Dermatological	0 (0)	0 (0)	na	0.05 (0.21)	0.04 (0.19)	0.86
Gastrointestinal	0.15 (0.37)	0.32 (0.47)	0.06	0.15 (0.36)	0.27 (0.45)	0.14
Headache, malaise	0.15 (0.37)	0.2 (0.41)	0.57	0.11 (0.31)	0.25 (0.44)	0.05
Musculoskeletal	0.25 (0.44)	0.48 (0.51)	0.03	0.3 (0.46)	0.32 (0.47)	0.79
Neurological	0 (0)	0.08 (0.28)	0.04	0.11 (0.31)	0.15 (0.36)	0.58
Respiratory	0.18 (0.39)	0.14 (0.35)	0.63	0.13 (0.34)	0.15 (0.36)	0.82
Sensory	0.08 (0.27)	0.16 (0.37)	0.22	0.09 (0.28)	0.23 (0.43)	0.04
Sleep	0.18 (0.39)	0.32 (0.47)	0.12	0.15 (0.36)	0.25 (0.44)	0.20
Urogenital	0 (0)	0 (0)	na	0.03 (0.15)	0.06 (0.23)	0.38
Any symptom	0.55 (0.51)	0.79 (0.42)	0.02	0.42 (0.5)	0.69 (0.47)	0.01

*na, non-applicable.*

We compared the presence of reported symptoms in children and adults living in the same household, to assess if presence of symptoms in adults may have affected the children (Table 5). Overall, there was a very low correlation at both follow-up timepoints (Bootstrapped confidence interval at 1–3 months follow-up: −0.148, −0.054; at 6–9 months follow-up: −0.078, 0.285).

**TABLE 5:** Correlation of symptoms between children and adult family members in the same household.

Symptom group	1–3 months follow-up			6–9 months follow-up		
		
	Sample correlation	Bootstrapped confidence interval	Number of observations	Sample correlation	Bootstrapped confidence interval	Number of observations
Bleeding	0.184	(0.042, 0.325)	63	0.325	(0.2, 0.444)	22
Cardiovascular	0.121	(−0.027, 0.273)	85	0.201	(0.117, 0.314)	79
Dermatological	0.040	(−0.101, 0.186)	85	0.172	(0.002, 0.318)	79
Gastrointestinal	0.011	(−0.127, 0.146)	85	0.159	(0.075, 0.226)	79
Headache, malaise	−0.028	(−0.046, −0.014)	63	0.062	(0.084, 0.217)	79
Musculoskeletal	−0.035	(−0.059, −0.019)	85	−0.052	(−0.075, −0.028)	79
Neurological	−0.089	(−0.129, −0.042)	85	−0.079	(−0.125, −0.045)	79
Respiratory	−0.094	(−0.129, −0.058)	85	−	−	22
Sensory	−	−	63	−	−	79
Sleep	−	−	85	−	−	79
Urogenital	−	−	85	−	−	22
Any symptom	−0.091	(−0.148, −0.054)	85	0.086	(−0.078, 0.285)	79

*Confidence intervals were calculated using bootstrap (observations are sampled 200 times with replacement; mean and 95% CI are calculated for 10,000 repetitions).*

In a multivariate regression analysis for both adults and children, to assess main factors related with the development of PASC, gender was the only significant risk factor, with risk for persisting symptoms higher in females amongst adults (Table 6).

**TABLE 6:** Evaluation of risk factors using logistic regression.

Factors	Model 1		Model 2		Model 3	
			
	Children (*n* = 66)		Children and adults (*n* = 375)		Children and adults (*n* = 634)	
			
	Estimate (95% CI)	*p*-value	Estimate (95% CI)	*p*-value	Estimate (95% CI)	*p*-value
Age (years)	−0.001 (−0.130, 0.128)	0.983	0.002 (−0.010, 0.014)	0.740		
Age range (10−17)					0.377 (−0.150, 0.904)	0.161
Age range (18−30)					0.851 (−0.090, 1.792)	0.076
Age range (30−40)					1.575 (0.893, 2.257)	0.000
Age range (40−50)					1.865 (1.304, 2.426)	0.000
Age range (50−60)					2.184 (1.525, 2.843)	0.000
Age range (60+)					1.168 (−0.398, 2.734)	0.144
Gender	−0.752 (−1.775, 0.271)	0.15	−0.839 (−1.264, −0.414)	0.000	−0.583 (−0.944, −0.222)	0.002
Comorbidity	0.290 (−0.778, 1.358)	0.595				
Hospitalisation	0.316 (−2.571, 3.203)	0.83	0.675 (−0.036, 1.386)	0.063		
PASC in adults in same household	0.031 (−1.230, 1.362)	0.963				

*PASC, post-acute sequelae of COVID-19; SE, standard error.*

Children and adults affected by one or more persistent symptoms reported lower overall health compared to before SARS-CoV-2 infection, although the differences were more pronounced in adults (Table 7). Moreover, parents reported changes in emotional behaviours, social relationships, and activity levels in a significant number of their children, mostly perceived by the parents as due to the pandemic (Supplementary Table 4).

**TABLE 7:** Changes in wellbeing and impact on daily activities among children with confirmed SARS-CoV-2 infection compared with before infection.

Impact on daily activities	1–3 months assessment		6–9 months assessment	
		
	Pre-SARS-CoV-2 infection	Post-SARS-CoV-2 infection	Pre-SARS-CoV-2 infection	Post-SARS-CoV-2 infection
**Children (≤18 years old) *n* (%)**				
Personal care	92/92 (100)	88/92 (96)	26/26 (100)	23/26 (89)
Daily activities	92/92 (100)	92/92 (100)	26/26 (100)	26/26 (100)
Anxiety	85/92 (93)	82/92 (90)	24/26 (93)	23/26 (89)
Breathlessness	92/92 (100)	76/92 (83)	26/26 (100)	25/26 (97)
Vision problems	91/92 (99)	91/92 (99)	24/26 (93)	23/26 (89)
Hearing loss	92/92 (100)	91/92 (99)	26/26 (100)	23/26 (89)
Difficulty moving	92/92 (100)	92/92 (100)	26/26 (100)	26/26 (100)
Lack of focus and concentration	88/92 (96)	89/92 (97)	25/26 (97)	25/26 (97)
No Language difficulties	87/92 (95)	92/92 (100)	26/26 (100)	26/26 (100)
**Adults**				
Personal care	101/101 (100)	92/101 (92)	106/107 (100)	90/107 (85%)
Daily activities	101/101 (100%)	98/99 (99%)	106/107 (100%)	104/107 (98%)
Anxiety	91/101 (91%)	76/101 (76%)	92/107 (86%)	86/107 (81%)
Breathlessness	100/101 (100%)	69/101 (69%)	106/107 (100%)	84/107 (79%)
Vision problems	97/101 (97%)	91/101 (91%)	100/107 (94%)	94/107 (88%)
Hearing loss	98/101 (98%)	91/101 (91%)	104/107 (98%)	94/107 (88%)
Difficulty moving	98/101 (98%)	95/101 (95%)	104/107 (98%)	102/107 (96%)
Lack of focus and concentration	101/101 (100%)	90/101 (90%)	105/107 (99%)	91/107 (86%)
Personal care	101/101 (100%)	82/101 (82%)	105/107 (99%)	91/107 (86%)
No Language difficulties	101/101 (100%)	98/101 (98%)	106/107 (100%)	103/106 (98%)

## Discussion

To our knowledge, this is the first study investigating the presence of PASC in a cohort of adults and children living in the same household. We found that one-third of children and two-thirds of adults had persistent symptoms at the time of the follow-up at 1–3 months after diagnosis, while at 6–9 months follow-up the prevalence of children with ongoing symptoms dropped to about one-in-ten. Of the children with SARS-CoV-2 confirmed infection, 16.7 and 5.1% showed signs of multi-system impact with two or more categories of persistent symptoms at 1–3 and 6–9 months follow up, respectively, many with impact on daily activities. The spectrum of PASC in adults was more frequent, lasted longer and was more severe, many impacted by signs of multi-system involvement. Age was associated with higher risk of prolonged symptomatology. Female gender was identified as a risk factor for PASC in adults, but not in children.

Although many children experienced symptoms, such as fatigue, muscle and joint pain, sleep and respiratory problems up to 3 months post-diagnosis, the prevalence was significantly lower at 6–9 months follow-up. The trend is in line with a recent, large paediatric study in Russia that found a steady decline in the symptom prevalence over time (9). While at 1–3 months the children with laboratory confirmed SARS-CoV-2 infection had a significantly higher probability of experiencing symptoms compared with those that tested negative, this difference was not significant at 6–9 months. This is in line with a recent study from Switzerland (14). This observation can have different explanations, it is possible that some symptoms such as headache, and sleep problems are common amongst this age group, which may be driven by several mechanisms including external factors and psychological ones (22). This is in line with the parents in this study mainly attributing their childrens psychosocial symptoms to the overall pandemic situation and restrictions.

The aetiology behind different clusters of PASC is still unknown. A standardised collection of self-reported symptoms can be a sensitive but not-specific tool for defining PASC. According to mounting evidence, PASC is a complex, multisystem disease where inflammatory and vascular defects, and “ghost virus” particles, can play a significant role (23). Our study adds to the evidence that a case definition for PASC will probably require evidence of multisystem involvement with impact on functioning. In our study we highlight the presentation of PASC by age, gender and several potential functional systems involved. This complex clinical picture is in line with aligned studies from Russia (9) and the United Kingdom (19). The subset of children, particularly those with multisystem involvement lasting for more than 3 months, requires further diagnostic assessments to forward our understanding into aetiology and risk factors for severe PASC. This information is essential to inform how to treat and, prevent longer term complications.

Overall, adults experienced more symptoms, more frequently and for a longer time. The differences were particularly relevant when children and adults were compared at 6–9 months follow-up after acute SARS-CoV-2 infection, where adults had significantly lower probability of full recovery and had more cardiovascular, respiratory and neurological symptoms. Moreover, adults with confirmed SARS-CoV-2 infection had a statistically significant higher rate of symptoms when compared to the negative control cohort. These findings are in line with another study that identified that adults can experience an average of 56 symptoms, across nine organ systems at 7 months post-COVID-19 onset, and that symptoms can be relapsing and triggered by exercise, physical or mental activity and stress (24). Although the pathogenesis of PASC is not well defined, it is interesting that our documentation of more severe PASC presentations in adults compared to children is in line with more severe acute diseases in older people. These findings may provide clues on the pathogenesis of PASC and suggest that since differences in the immune responses may explain differences during acute infection (25, 26), similar differences can be implicated in more severe adult PASC. For example, age-related decline and dysregulation of immune function, such as immunosenescence and inflammaging, can explain a gradient of severity of PASC as age increase (27). Also, younger children have more frequently an asymptomatic disease which, in previous studies, have been associated with lower virus load and faster virus clearance (28, 29). This can support the hypothesis that asymptomatic children have lower risks of developing PASC, although in our study only 20 children were asymptomatic limiting the possibility of a statistical analyses aimed at defining this. In addition, age-related differences ACE2 and TMPRSS2 expression profiles at the epithelial sites of the lung and skin, and also alterations of expression of these genes in the PBMCs and T cells from healthy children and adults, can explain why PASC in adult is more severe and involve more organs (30). Important to know that, however, ACE2 expression levels are mostly detailed in animal studies.

An important finding of our study is that we found no correlation between PASC symptoms in adults and their children living in the same household. Considering that there is a narrative that PASC symptoms may be of psychological origin, in adults and children, we would have expected that children seeing suffering adults may have had a higher risk of showing similar problems. However, contrary to this hypothesis, the correlations found were extremely weak, indirectly supporting that PASC have a predominantly organic background, determined by complex and still poorly understood interactions between the virus and the host.

Even if the impact of PASC identified in adults did not directly impact on the children in this study, it may still have significant long-term effects in families with parents with long term PASC. Children are a fragile population that may be severely impacted by others suffering, such as their parents. A recent global estimate found that 1,134,000 children (95% credible interval 884,000–1,185,000) have experienced the death of primary caregivers due to the pandemic, including at least one parent or custodial grandparent, with the highest rates in low-to-middle income countries (31). The authors defined the orphanhood and caregiver deaths as a “hidden pandemic” resulting from COVID-19-associated deaths. However, these estimates only consider the burden of parental mortality on child wellbeing, not the impact of chronic sequelae that can lead to different degrees of new or worsened disability (19). In the United Kingdom alone, an estimated 2 million people are suffering from PASC (32), and a recent study showed that among 3762 adults with COVID-19, >91% of patients required more than 35 weeks to recover, and 77.5% reported impact on their ability to work or return to work (24). Although this aspect has not yet been studied, anecdotal reports show that adults severely affected by PASC, with fatigue and muscle weakness, are having to rely on their children to help care for them. It is therefore reasonable to suspect that the physical, mental, and also economic impact of PASC in adults may have implications on their ability to care for and support their children and families, and in countries with insufficient social care structures, such as the United Kingdom, children will have to help support their families. So far, public health interventions have mainly focussed on acute COVID-19 outcomes without considering long-term outcomes and impact.

This study is not without limitations. Firstly, the study is based on self-reported symptoms, and did not include further clinical, laboratory and radiologic investigation to validate findings objectively due to the ongoing pandemic and its resource limitations. Secondly, the number of patients included was relatively small, which may impact on the results and its generalisability.

This also limited in our ability to have enough data to address how severity or clinical phenotypes of acute disease, length of COVID-19, and different variants could affect the future risk of developing PASC; future studies should take into account a deeper characterisation of acute disease and probable variants causing acute infection and analyse how this impacts on longer term outcomes. Thirdly, we cannot rule out that household contacts that tested negative through nasopharyngeal tests are false negative results, since serologic assessments were not made at time of the survey. However, this risk was minimised by testing contacts twice, after exposure and 10 days later. Finally, we did not assess the ability of parents with PASC to care for their children and if their caring role further exacerbated their PASC symptomatology or wellbeing. Nevertheless, this study provides important data to characterise PASC in different age groups and risk factors for PASC by age and gender and compared to controls matched by geography, time and pandemic restrictions. This is to our knowledge the first study characterising and comparing PASC in children and adults and matched household controls. This study also highlights the importance of developing dedicated Post COVID clinics for both adults and children in order to offer appropriate and comprehensive clinical care, and not only focus on mental health. Specifically, a 3 months cut-off of “wait and see” approach seems reasonable, since most children improved during this period, while those with persisting symptoms should enter a dedicated personalised approach. We have presented our local practice in a previous study (11, 33).

## Conclusion

Our study found that children can experience PASC, but at a lower frequency and less severe compared with adults. In children, a majority of cases resolved spontaneously within 6 months from SARS-CoV-2 infection. However, a subset of children experienced a cluster of multiple systems symptoms, and further characterisation and assessment of these children is of vital importance to understand how to prevent and treat them to improve long-term outcomes. Although the prevalence of children affected by PASC were lowered compared to adults, a recovery time of more than 3 months may have adverse impacts on education and relationships, and as our study found further impacted by the pandemic. In addition, the high burden of PASC experienced by adult household members, with an impact on quality of life, including ability to work, may further indirectly affect the wellbeing of their children. These findings require further studies to understand how PASC impacts on families health and well being long-term. Our findings also highlight an urgent need to further characterise PASC syndrome in all age groups and interventional studies into treatment and prevention. This represents an urgent health priority given the potential direct and indirect enormous implications on a global health perspective.

## Data Availability Statement

The raw data supporting the conclusions of this article will be made available by the authors, without undue reservation after request to the corresponding author.

## Ethics Statement

The studies involving human participants were reviewed and approved by the Fondazione Policlinico Universitario A. Gemelli IRCCS. Written informed consent to participate in this study was provided by the participants’ legal guardian/next of kin. Written informed consent was obtained from the minor(s)’ legal guardian/next of kin for the publication of any potentially identifiable images or data included in this article.

## Members of the FIMP-Roma Study Group

Ilaria Sani, Giovanna La Cava, Serenella Castronuovo, Isabella Capodici, Ermenia Zirletta, Loredana Costabile, Di Martino, Lorenza Arnaboldi, Maria Concetta Carbone, Rosella Sebastianelli, Cristina Ciuffo, Donatella Marano, Cinzia Grassi, Immacolata La Bella, and Luciano Sozio.

## Author Contributions

DB and PV conceptualised the study. DM and LS coordinated the development of the study survey. DB, DM, and LS drafted the final version of the manuscript. All authors contributed with data collection and read and approved the last version of the manuscript.

## Conflict of Interest

The authors declare that the research was conducted in the absence of any commercial or financial relationships that could be construed as a potential conflict of interest.

## Publisher’s Note

All claims expressed in this article are solely those of the authors and do not necessarily represent those of their affiliated organizations, or those of the publisher, the editors and the reviewers. Any product that may be evaluated in this article, or claim that may be made by its manufacturer, is not guaranteed or endorsed by the publisher.
