# Supplementary material for: Post-COVID Condition in Adults and Children Living in the Same Household in Italy: A Prospective Cohort Study Using the ISARIC Global Follow-Up Protocol
Source: Front Pediatr. 2022 Apr 21;10:834875. doi: 10.3389/fped.2022.834875 (PMC9070551; doi:10.3389/fped.2022.834875)
Supplement: Supplementary file 1 [file Data_Sheet_1.docx]

**Supplementary Table S1** - Comorbidities and risk factors among all positive children included in the study

| **Comorbidity** | **Frequency** | **Number of observations** |
| --- | --- | --- |
| Prematurity | 12% | 236 |
| Neurological | 7% | 243 |
| Neurodisability | 1% | 242 |
| Heart diseases | 2% | 243 |
| Respiratory diseases | 7% | 240 |
| Asthma | 3% | 243 |
| Allergic rhinitis, hay fever | 3% | 241 |
| Food allergy | 2% | 243 |
| Atopic dermatitis eczema | 7% | 243 |
| Skin problems | 1% | 241 |
| Gut problems | 2% | 240 |
| Haematology | 0% | 243 |
| Oncology | 0% | 243 |
| Immune system diseases | 1% | 243 |
| Genetic conditions | 0% | 242 |
| Diabetes | 0% | 243 |
| Endocrine | 0% | 243 |
| Renal Kidney problems | 0% | 242 |
| Excessive weight and obesity | 2% | 243 |
| Malnutrition | 0% | 242 |
| Rheumatology | 0% | 240 |
| Depression | 0% | 123 |
| Anxiety | 1% | 123 |
| HIV | 0% | 120 |

**Supplementary Table S2** **- Symptoms experienced in the first 14 days of COVID-19 illness by PCR positive children**

| **Symptom** | **1-3 months from COVID**  **n (%)** | **6-9 months from COVID**  **n (%)** |
| --- | --- | --- |
| Cough | 45 / 88 (51,1) | 53 / 103 (51,5) |
| Shortness of breath | 4 / 88 (4,5) | 1 / 104 (1) |
| Fatigue | 24 / 88 (27,3) | 23 / 104 (22,1) |
| Pain on breathing | 1 / 88 (1,1) | 1 / 104 (1) |
| Chest pain | 1 / 88 (1,1) | 1 / 104 (1) |
| Loss or disturbed smell | 15 / 88 (17) | 21 / 104 (20,2) |
| Loss or disturbed taste | 16 / 88 (18,2) | 22 / 104 (21,2) |
| Runny nose | 24 / 88 (27,3) | 21 / 104 (20,2) |
| Headache | 24 / 88 (27,3) | 24 / 104 (23,1) |
| Muscle pain | 15 / 88 (17) | 18 / 104 (17,3) |
| Abdominal pain | 8 / 88 (9,1) | 4 / 104 (3,8) |
| Vomiting | 4 / 88 (4,5) | 2 / 104 (1,9) |
| Diarrheoa | 11 / 88 (12,5) | 4 / 104 (3,8) |
| Confusion | 11 / 88 (12,5) | 4 / 104 (3,8) |
| Brain fog | 1 / 88 (1,1) | 1 / 104 (1) |
| No symptoms | 18 / 90 (20) | 14 / 105 (13,3) |
| Other | 18 / 90 (20) | 14 / 105 (13,3) |

**Supplementary Table S3** - Symptom persistence among children and adults with PCR confirmed SARS-CoV-2 infection interviewed at both follow-up timepoints

| **Symptom group** | **Children** | | | **Adults** | | |
| --- | --- | --- | --- | --- | --- | --- |
|  | **1-3 months**  **number of patients** | **6-9 months**  **number of patients** | **p-value** | **1-3 months**  **number of patients** | **6-9 months**  **number of patients** | **p-value** |
| Bleeding | 0 | 0 | - | 0 | 0 | - |
| Cardiovascular | 0 | 0 | - | 1 | 2 | 0,32 |
| Dermatological | 4 | 0 | 0,04 | 0 | 0 | - |
| Gastrointestinal | 11 | 0 | 0,00 | 10 | 11 | 0,57 |
| Headache, malaise | 11 | 0 | 0,00 | 7 | 2 | 0,06 |
| Musculoskeletal | 4 | 0 | 0,04 | 10 | 8 | 0,32 |
| Neurological | 2 | 0 | 0,16 | 3 | 1 | 0,16 |
| Respiratory | 2 | 0 | 0,16 | 3 | 3 | 1,00 |
| Sensory | 4 | 0 | 0,04 | 4 | 4 | 1,00 |
| Sleep | 0 | 0 | - | 8 | 7 | 0,32 |
| Urogenital | 1 | 0 | 0,32 | 0 | 0 | - |
| Any symptom | 26 | 0 | 0,00 | 22 | 19 | 0,26 |

**Supplementary Table S4** - Changes in activities and behaviour amongst children in total and the proportion attributed to Covid-19 illness

|  | **1-3 months**  **Assessment**  **n (%)** | | **Proportion attributed to PASC**  **n (%)** | **6-9 months**  **Assessment**  **n (%)** | **Proportion attributed to PASC**  **n (%)** | |
| --- | --- | --- | --- | --- | --- | --- |
| Eating more | 8/174 (4,6) | | 8 / 8 (100) | 6 / 136 (4,4) | | 6 / 6 (100) |
| Sleeping less | | 33/174 (19) | 29 / 33 (87,9) | 21 / 136 (15,4) | 19 / 21 (90,5) | |
| Reduced physical activity | 95/174 (54,6) | | 57 / 95 (60) | 55 / 136 (40,4) | 37 / 55 (67,3) | |
| Increased fatigue | 38 / 174 (21,8) | | 0 / 38 (0) | 20 / 136 (14,7) | 0 / 20 (0) | |
| Less frequent communication with friends in-person | 70 / 172 (40,7) | | 56 / 70 (80) | 51 / 136 (37,5) | 40 / 51 (78,4) | |
| Less frequent communication with friends remotely | 2 / 172 (1,2) | | 2 / 2 (100) | 5 / 135 (3,7) | 3 / 5 (60) | |
| Spending less time outside | 87 / 173 (50,3) | | 57 / 87 (65,5) | 64 / 136 (47,1) | 45 / 64 (70,3) | |
| Impacting negatively on: |  | |  |  |  | |
| Connectedness with others | 2 /135 (1,5) | | 2 / 2 (100) | 6 / 42 (14,3) | 6 / 6 (100) | |
| Emotions | 49 / 136 (36) | | 38 / 49 (77,6) | 20 / 42 (47,6) | 15 / 20 (75) | |
| Behaviour | 26 / 136 (19,1) | | 25 / 26 (96,2) | 12 / 42 (28,6) | 10 / 12 (83,3) | |
| Relationships | 18 / 136 (13,2) | | 17 / 18 (94,4) | 9 / 41 (22) | 8 / 9 (88,9) | |

Abbreviations: PASC:
